# A Field-Tailored Reverse Transcription Loop-Mediated Isothermal Assay for High Sensitivity Detection of *Plasmodium falciparum* Infections

**DOI:** 10.1371/journal.pone.0165506

**Published:** 2016-11-08

**Authors:** Sylvie Kemleu, Dylan Guelig, Carole Eboumbou Moukoko, Estelle Essangui, Steven Diesburg, Abas Mouliom, Bernard Melingui, Jeanne Manga, Christiane Donkeu, Annie Epote, Gaëtan Texier, Paul LaBarre, Robert Burton, Lawrence Ayong

**Affiliations:** 1 Malaria Research Unit, Centre Pasteur du Cameroun, Yaoundé, Cameroon; 2 PATH, Seattle, Washington, United States of America; 3 Faculty of Medicine and Pharmaceutical Sciences, University of Douala, Douala, Cameroon; 4 Faculty of Sciences, University of Douala, Douala, Cameroon; 5 Hematology Unit, Centre Pasteur du Cameroun, Yaoundé, Cameroon; 6 Public Health & Epidemiology Unit, Centre Pasteur du Cameroun, Yaoundé, Cameroon; Université Pierre et Marie Curie, FRANCE

## Abstract

Highly sensitive and field deployable molecular diagnostic tools are critically needed for detecting submicroscopic, yet transmissible levels of malaria parasites prevalent in malaria endemic countries worldwide. A reverse transcription loop-mediated isothermal amplification (RT-LAMP) assay was developed and evaluated in comparison with thick blood smear microscopy, an antigen-based rapid diagnostic test (RDT), and an in-house RT-PCR targeting the same RT-LAMP transcript. The optimized assay detected *Plasmodium falciparum* infections in as little as 0.25ng of total parasite RNA, and exhibited a detection limit of 0.08 parasites/ μL when tested directly on infected whole blood lysates, or ~0.0008 parasites/ μL when using RNA extracts. Assay positivity was observed as early as eight minutes from initiation of the RT-LAMP and in most cases the reaction was complete before twenty minutes. Clinical evaluation of the assay on 132 suspected malaria cases resulted in a positivity rate of 90% for RT-LAMP using extracted RNA, and 85% when using whole blood lysates. The positivity rates were 70% for *P*. *falciparum*-specific RDT, 83% for RT-PCR, and 74% for thick blood smear microscopy (Mean parasite density = 36,986 parasites/ μL). Concordance rates between the developed RT-LAMP and comparator tests were greater than 75%, the lowest being with light microscopy (78%, McNemar’s test: P = 0.0002), and the highest was with RT-PCR (87%, McNemar’s test: P = 0.0523). Compared to reference RT-PCR, assay sensitivity was 90% for RT-LAMP on whole blood, and 96% for RT-LAMP using corresponding RNA extracts. Electricity-free heaters were further developed and evaluated in comparison with a battery-operated isothermal amplification machine for use with the developed test in resource-limited settings. Taken together, the data highlight the benefits of targeting high abundant RNA transcripts in molecular diagnosis, as well as the potential usefulness of the developed RT-LAMP-assay in malaria diagnosis in low to high parasite density settings.

## Introduction

Malaria is a preventable and treatable parasitic infection with historically broad endemicity globally[1]. Today, the disease is mostly confined to parts of Africa, Southeast Asia and Latin America, where an estimated 3.2 billion people are at risk of being infected by the causative agents. At least five *Plasmodium* species (*P*. *falciparum*, *P*. *vivax*, *P*. *malariae*, *P*. *ovale*, *and P*. *knowlesi*) are known to cause malaria in humans, the most virulent being *P*. *falciparum*. As a result of intense malaria interventions implemented at an unprecedented scale in the last century, malaria burdens have declined significantly in most endemic countries leading to an overall estimate of just 214 million cases and 438,000 deaths in 2015 (*WHO*: *World Malaria Report 2015*). The epidemiology of malaria has also changed in several endemic countries, shifting the focus of some intervention programs to active detection and elimination of the parasite reservoirs that consist mostly of asymptomatic malaria cases [2–7]. With prospects of mass testing and treatment being considered worldwide[8], there is a crucial need for extremely sensitive and field applicable diagnostic systems to aid the ongoing efforts against malaria. Simple, rapid, and ultra-sensitive diagnostic tests are also needed for pre-screening of blood samples particularly in residents or visitors to malaria endemic zones before transfusion, and for identifying asymptomatic malaria in pregnant mothers to avoid congenital transmission[9–16].

Malaria diagnosis is currently conducted by light microscopy (thick or thin blood smears), by using one of several existing antigen-based lateral flow immunological tests (commonly referred to as rapid diagnostic tests or RDTs),or using nucleic acid amplification-based tests (NAATs) such as polymerase chain reaction (PCR) or isothermal amplification techniques [17, 18]. Of these, light microscopy remains the gold standard given that a positive result, if accurately interpreted, proves an active infection with malaria parasites. Light microscopy, however, is not sufficiently sensitive for use in cases of low density parasite carriage given its detection limit of approximately 100 parasites/μL for most mid-level microscopists[17, 19]. Additionally, the technique is labor-intensive, operator-dependent, and difficult to perform on a large scale. Malaria RDTs represent important alternatives to light microscopy as they are simple to use, rapid(assay time of 15 to 20 minutes), and requiring no electricity during the assay [20, 21]. Major limitations with the existing RDTs lie with their high degree of false-negatives in some malaria elimination settings, occurring due to their high detection limits (>100 parasites/μL),high frequency of target sequence deletion in certain regions, as well as the so-called "hook” or “prozone” effect occurring in high antigenaemic cases[21–29]. False positive outcomes are also common due to antibody cross-reactivity to blood rheumatoid factor and other tropical infections [30–33], and because of the long median clearance times of approximately 28 and 7 days for the frequently targeted *P*. *falciparum* histidine-rich protein II(*Pf*HRP2) and *Plasmodium* species-specific lactate dehydrogenase (pLDH) antigens, respectively[34, 35].

Nucleic acid amplification techniques represent the most sensitive diagnostic approaches for malaria to date, providing the levels of sensitivity that are required for asymptomatic *Plasmodium* infection detection in malaria elimination settings [18, 36–39]. Of the existing molecular approaches, those targeting high-abundant RNA transcripts and multi-copy DNA sequences provide the highest analytical sensitivity ranging between 0.002 and5 parasites/ μL of blood, compared to 0.7 to 10 parasites/ μL limits of detection for other DNA-based approaches[40–44]. The RNA-based methods include traditional and quantitative reverse transcriptase PCRs and nucleic acid sequence-based amplification(NASBA) methods, which rely on detection of the parasite 18S rRNA transcript in purified RNA samples [44, 45]. The field application of the above RNA amplification techniques, however, is limited by the requirement for highly trained personnel, electrified instrumentation, and large sample volumes (approximately 50 μL of paper filter-spotted blood or >200 μL of tube collected blood)[46, 47], as well as their susceptibility to cross contamination and to common PCR inhibitors[18].

Reverse transcription loop-mediated isothermal amplification (RT-LAMP) provides the level of sensitivity needed for asymptomatic malaria diagnosis in low-to-moderate infection scenarios, and the simplicity and cost-effectiveness needed for use in resource-limited environments. Generally, LAMP is a nucleic acid amplification technique that is based on the use of strand displacement polymerases, such as *Bst* polymerase, to amplify target sequences under isothermal conditions of about 65°C, depending on the enzyme version and target DNA [48, 49]. The method necessitates 4 or 6 oligonucleotide primers recognizing 6 or 8 distinct sequence regions of the target, thereby eliminating the non-specific amplification of closely related nucleic acid sequences[48]. For RNA targets, a second enzyme (often AMV reverse transcriptase) is added to generate the required cDNA template prior to the LAMP reaction. Recent advances in LAMP enzyme technology, however, have led to the discovery and commercialization of single-enzyme kits such as OmniAmp (Lucigen Corporation, USA), *Bst* 3.0 (New England Biolabs, USA),and GspSSD LF (OptiGene, UK) polymerases, which exhibit both reverse transcription and strand displacement polymerase activities, thereby facilitating the development of more rapid and sensitive RT-LAMP systems.

To further facilitate the field use of isothermal amplification techniques in resource-limited settings, PATH developed an inexpensive, highly efficient, and electricity-free non-instrumented nucleic acid amplification (NINA) system that is based on an exothermic reaction between common salt and Mg-Fe mechanical alloy [50–55]. We describe here the development of a novel RT-LAMP technique that is based on detection of a high-abundant *Plasmodium falciparum* transcript in parasite RNA extracts or infected whole blood lysates using commercially available single enzyme systems and the NINA approach.

## Materials and Methods

### Clinical samples

Ethical clearance and administrative approval of the study were obtained from the Cameroon National Ethics Committee (N°2014/05/458/L/CNERSH/SP) and Ministry of Public Health, respectively. Written informed consent was further obtained from each patient/guardian prior to sample collection, or use of leftover blood samples from microscopically confirmed *Plasmodium* infected persons identified from routine laboratory testing of suspected malaria cases at the Hematology Unit of Centre Pasteur Cameroon. The parasite density (parasites per μL of whole blood) in each infected sample was determined by thick blood smear microscopy, assuming a white blood cell count of 8000/ μL blood volume, whereas confirmation of the infecting *Plasmodium* spp was by thin blood smear microscopy. Samples were further characterized using the SD Bioline Malaria Ag *P*. *f*./Pan-*Plasmodium* rapid diagnostic test (Standard Diagnostics) for detection of *Plasmodium falciparum*-specific histidine-rich protein 2 (PfHRP2) and/orpan-*Plasmodium*-specific lactate dehydrogenase (pan-pLDH) antigens.

### RNA extraction

Total RNA was extracted from freed parasite pellets by using a Trizol-based method[56–58]. Briefly, 2 mL of each buffy coat-free blood sample were saponin-treated for five minutes on ice, and centrifuged at 2000 rpm to collect freed parasites. All other RNA extraction steps were as per the manufacturer’s instructions using 500 μL of Tri reagent (Invitrogen) to solubilize the parasite pellet. The precipitated RNA was then dissolved in 50 μL of DEPC-treated water, and the content quantified using a Nanodrop 2000 spectrophotometer.RDT and microscopically negative samples were also subjected to the above RNA extraction protocol, and the resulting extraction product analyzed by both RT-LAMP and RT-PCR assays.

### RT-LAMP primers and assay conditions

Primers were designed targeting specific segments of the high-abundant *Plasmodium falciparum* Exported protein 1 (*PfExp1*, PF3D7_1121600) open-reading frame using the licensed LAMP Designer software version 1.12 (OptiGene, UK). The obtained primer sequences were further subjected to Basic Local Alignment Search Tool (BLAST) analyses at NCBI GenBank to confirm specificity to malaria parasites. Orthologues of the *PfExp1* gene were either retrieved from the *Plasmodium* genome database (PVX_091700 and PKNH_0919300) or requested from the Wellcome Trust Sanger Institute malaria genome project (*P*. *ovale* and *P*. *malariae*) and compared by Clustal W2 sequence alignment[59]. Selected primer sets were screened for positive amplification of a parasite RNA sample using the Genie II real-time fluorescence-based isothermal amplification machine (OptiGene, Ltd, UK) and a temperature gradient of 62°Cto70°C. The real-time amplification curves were then scored using the derived time-to-peak amplification durations that were obtained using the Genie II software version 1.27. Primer sets and temperature conditions resulting in the shortest time-to-result durations, and no positive signals in no-template control wells were selected for further optimization of the RT-LAMP protocol.

The optimized RT-LAMP reaction consisted of1.6 μM forward (F3) and backward (B3) primers, 0.8 μM forward-inner (FIP) and backward-inner (BIP) primers, and 0.4 μM loop forward (LF) and loop backward (LB) primers in a total reaction volume of 25 μL. Upon addition of 2.5 μL of the RNA sample and 15 μL of reconstituted enzyme mix (ISO-DR001, OptiGene), the reaction tubes were immediately transferred into the Genie II amplifier and maintained at 68°C for up to 60 minutes. Reconstitution of the lyophilized dual activity enzyme in the ISO-DR001 kit was as instructed by the manufacturer (OptiGene, UK) using a proprietary buffer system that presumably contains a DNA intercalating dye that is detectable by the Genie II real-time amplifier (OptiGene, UK). An RT-LAMP inactivation/annealing step of 98–50°C with rampat 0.1°C per minute was included, and the derived melting curves were used to verify the reaction specificity. The RT-LAMP products were also routinely analyzed by agarose gel electrophoresis (1.2% gels) to verify the expected laddering pattern of positive LAMP reaction products.

### RT-LAMP assays with whole blood lysates

As a simple and rapid sample-processing procedure, 10μL of *P*. *falciparum*-infected whole blood samples were initially diluted in lysis buffer (1/2, 2/5, 1/5 and 1/10 of 2X buffer or 1/10, 1/20 and 1/50 of 1X buffer), consisting of 10 mM Tris-buffered saline, pH 7.4,0.2% Triton X-100, and 0.1% bovine serum albumin, and left standing at room temperature for various lysis durations. The resulting lysates (2.5 μL) were added to 22.5 μL of RT-LAMP master mix and amplified as described above using the Genie II real-time amplifier. The sensitivity limit of the whole blood lysis method was also determined following serial dilution of microscopically (thin blood smear) confirmed *P*. *falciparum* mono-infected samples in uninfected blood prior to lysis and RT-LAMP analysis.

### RT-LAMP assays in electricity-free NINA heaters

Electricity-free NINA heaters were designed to maintain a constant temperature of 65–68°C for over 60 minutes following heat activation [60]. To activate the system, a pouch of Mg-Fe fuel was placed in the customized thermos, and 5 mL of 0.9% saline was added. The RT-LAMP tubes (maximum of twelve 0.2 mL PCR tubes per heater) in tube holders were placed in the heater (thermos), which was left at room temperature for up to 60 minutes. Gel Green solution (125X final concentration) was added before or after amplification in each reaction tube, and the resulting fluorescence was viewed using a hand-held UV-C flashlight (UVG-4, UVP Ltd) or a standard laboratory UV-A light box.

### Clinical evaluation of developed RT-LAMP assays

Clinical assessment of the developed RT-LAMP was in comparison with light microscopy, antigen-based RDT, and RT-PCR using blood samples from a total of 98 microscopy positive and 34 negative individuals. 2.5 μL extracted RNA (from standardized 2 mL whole blood samples) were tested by RT-LAMP using the Genie II amplifier or RT-PCR targeting the same *PfExp1*sequence. Similarly, 2.5μL of whole blood lysates (10 μL whole blood in 490 μL lysis buffer) were tested using the Genie II real-time amplifier or NINA heaters. The RT-PCR was performed using the RT-LAMP outer primers, F3 and B3, as forward and reverse primers, respectively. Briefly, 2.5 μL RNA samples were added to a reaction master mix comprising 0.2 μM each forward and reverse primer, and 12.5 μL of a One-step RT-PCR enzyme mix (AmpliTaq) in a total reaction volume of 25 μL. A film of mineral oil was then added to each tube and the RT-PCR performed using the following cycling conditions: 42°C for 10 minutes, and 35 cycles of 95°C for 15 seconds, 60°C for 10 seconds and 68°C for 60 seconds. The amplification products were further analyzed on a 1.2% agarose gel and scored as RT-PCR positive or negative depending on UV visualization of the expected band size.

## Results

### Diagnostic potential of the *PfExp1* RT-LAMP assay

**Fig 1:**
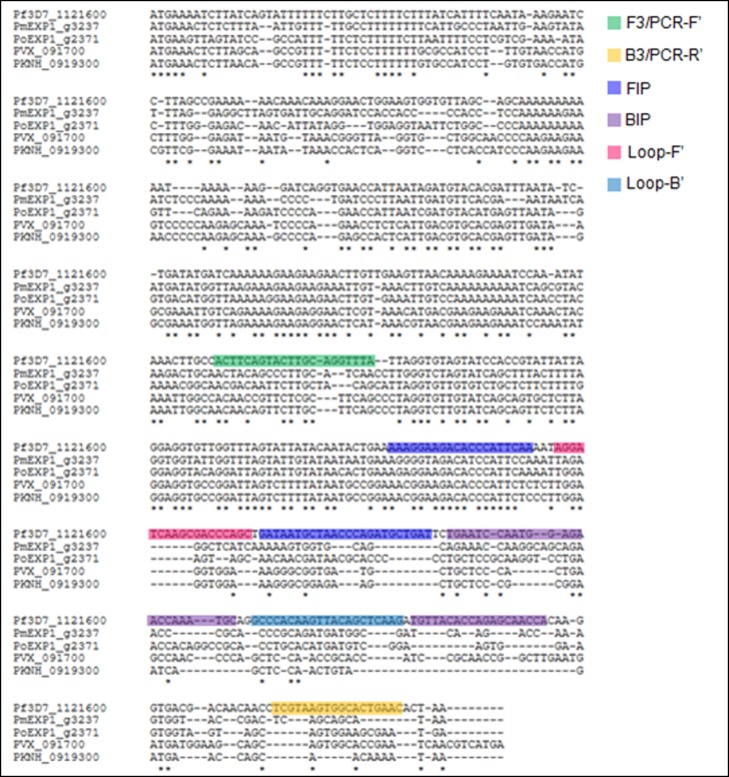
Sequence alignment of *PlasmodiumExp1* genes and RT-LAMP primer locations. Comparison of known human malaria *Plasmodium* exported protein 1 (*PfExp1*) open-reading frames reveal important differences in nucleotide sequences around the *PfExp1* primer sites. The full-length *P*. *vivax* (PVX_091700), *P*. *knowlesi* (PKNH_0919300) and *P*. *falciparum* (Pf3D7_1121600) exported protein 1 (*Exp1*) sequences were retrieved from PlasmoDB (www.plasmodb.org), whereas the *P*. *malariae* and *P*. *ovale* orthologues were obtained from the Wellcome Trust Sanger Institute. *Asterisks* indicate nucleotide bases common to all five *Plasmodium* species.

**Fig 2:**
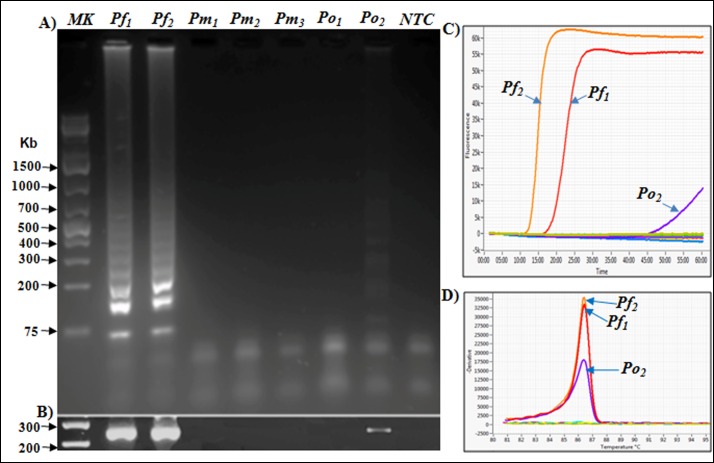
Species-specificity of the developed RT-LAMP and RT-PCR assays. Total RNA was extracted from microscopically confirmed *P*. *falciparum*-infected (*Pf*), *P*. *malariae*-infected (*Pm*), or *P*. *ovale*-infected (*Po*) blood samples, and 2.5μL extract were tested alongside a "no template" control (NTC) by both RT-LAMP and RT-PCR methods. **A)** Agarosegel (1.2%) analyses of the RT-LAMP products, showing strong positive amplification in *Pf*-positive RNA samples, and a weak positive banding pattern in one of two *P*. *ovale* samples (Po_2_). **B)** Agarosegel (1.2%) analyses of the RT-PCR products showing similar sample positivity profiles as with the RT-LAMP method. **C)** RT-LAMP amplification profiles showing rapid amplification signals in the identified *Pf* samples, and delayed amplification in the *Po2* reaction tube, suggesting presence of extremely low levels of target transcripts. **D)** Melting curves of all RT-LAMP reaction tubes, indicating that the same products were generated in each RT-LAMP positive tube.

**Fig 3:**
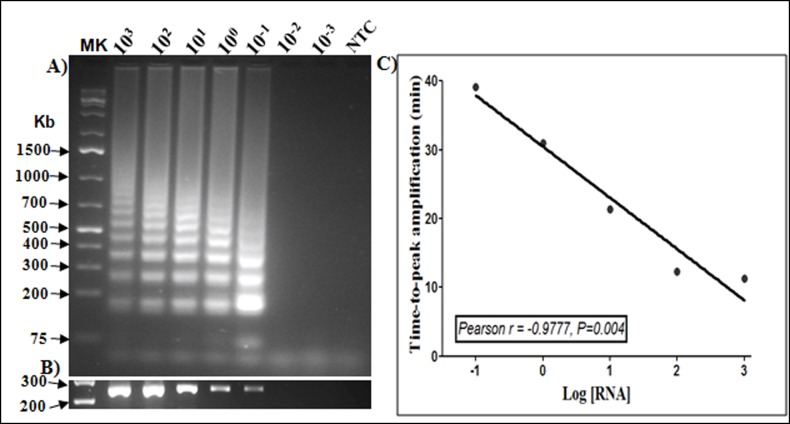
Detection limit of RT-LAMP and RT-PCR assay using total RNA extracts. Extracted total RNA from a *P*. *falciparum*-infected blood sample was serially diluted (10^3^to 10^-3^ng/μL) and 2.5μL tested by both RT-LAMP and RT-PCR methods. **A)** Agarose (1.2%) gel analyses and visualization of DNA laddering in RT-LAMP amplified samples. **B)** Agarose (1.2%) gel analyses of the *PfExp1* RT-PCR products, showing similarities in the detection limits of both RT-LAMP and RT-PCR methods. *Mk*: 1Kb DNA ladder, *NTC*: no-template control reaction tube, and numbers on each lane indicate the total RNA concentration (in ng/μL) in each tube prior to the assays.**C)** Linear regression analyses showing a significant inverse correlation between total RNA amounts and the time-to-peak amplification signal for the RT-LAMP method. Data is representative ofthe lowest limit of detection obtained from three replicate experiments at different diluted RNA concentration ranges.

**Fig 4:**
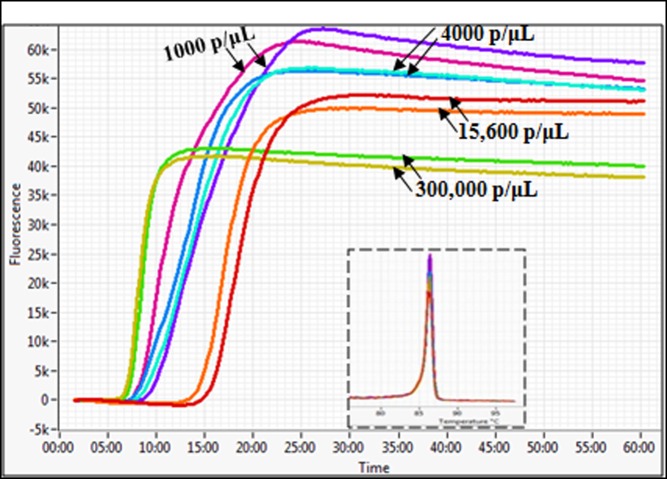
Reproducibility of the RNA extraction and RT-LAMP methods. RNA was repeatedly extracted from the same patient samples (2 ml whole blood), and 2.5 μL each extract were analyzed by RT-LAMP. The parasite density in each tested sample is indicated on the corresponding amplification profile. Both the 1000 and 4000 parasites/ μL samples were microscopically determined as *P*. *falciparum* mono-infected cases, whereas the 15,600 and 300,000 p/μL samples represented *P*. *falciparum*/*P*. *malariae* co-infections. Insert is the derived melting curve plot of all duplicate amplified samples, confirming that the same DNA products were obtained in each reaction tube.

The *PfExp1* target is a conserved *Plasmodium* gene that is abundantly expressed in all asexual blood forms of *Plasmodium* parasites (minimum expression percentile = 98%) [61–63]. To develop a molecular tool for high-sensitive detection of *P*. *falciparum* infections in clinical samples, we synthesized six specially designed RT-LAMP primers targeting a 258-bp fragment of the *PfExp1* transcript. Details of the *PfExp1* RT-LAMP primers and their sequence locations are provided in Table 1 and Fig 1, respectively. Sequence alignment of the *PfExp1* open-reading frame with orthologues in other *Plasmodium* species (*P*. *ovale*, *P*. *malariae*, *P*. *vivax*, and*P*. *knowlesi*, Fig 1) also revealed important differences at the RT-LAMP primer sites. To verify the species-specificity of the *PfExp1*-based RT-LAMP and RT-PCR methods, total RNA from microscopically confirmed *P*. *falciparum*, *P*.*malariae*or, *P*. *ovale* infected blood samples were tested by both methods. As shown in Fig 2A and 2B, both RT-LAMP and RT-PCR assays were positive for all two *P*. *falciparum*-infected samples, negative for all three *P*. *malariae* samples, and only weakly positive for one of two *P*. *ovale* samples. The real-time amplification profiles and derived melting curves are shown in Fig 2C and 2D, respectively, indicating that the same amplification products were obtained in all positive reaction tubes (i.e. same melting temperature of 86.5°C). The data also suggest that the lone weakly positive result of the *P*. *ovale* infected sample might represent an extremely low-density *P*. *falciparum* and *P*. *ovale* mixed infection. Both RT-LAMP and RT-PCR test methods detected *P*. *falciparum* infection in as little as 0.1 ng of total parasite RNA per μL sample (Fig 3A and 3B), indicating the equivalence of RT-LAMP with traditional RT-PCR in terms of test sensitivity limit. A high inverse correlation (Pearson r = -0.9777, P = 0.004) was also obtained between the resulting RT-LAMP peak amplification times and the input RNA amounts (Fig 3C), suggesting that the developed RT-LAMP might be useful in estimating the parasite density in infected samples. The same amplification profiles/peak amplification times were obtained when RT-LAMP was performed in duplicate using samples from the same infected individuals (Fig 4). This result establishes that the optimized RT-LAMP protocol is reproducible within the investigated parasite density range (1000–300,000 parasites/ μL). Together, the above data support ourconclusionsthatthedeveloped*PfExp1*-basedRT-LAMPissufficiently robust for use in high-sensitive detection of low-density *P*. *falciparum* infections.

**Table 1:** RT-LAMP primer sequences.

Targetedgene	Primer name	Primer sequence (5’ to 3’)
*PfExp1*	F3	ACTTCAGTACTTGCAGGTTTA
	B3	GTTCAGTGCCACTTACGA
	FIP	ATCAGCATCTGGGTTAGCATTATCAAAAGGAAGACACCCATTCAA
	BIP	TGAATCCAATGGAGAACCAAATGCGGTTGCTCTGGTGTAACA
	Loop F’	GCTGGGTCGCTTGATCCT
	Loop B’	GCCCACAAGTTACAGCTCAAG

### Performance of RT-LAMP assay on whole blood lysates

**Fig 5:**
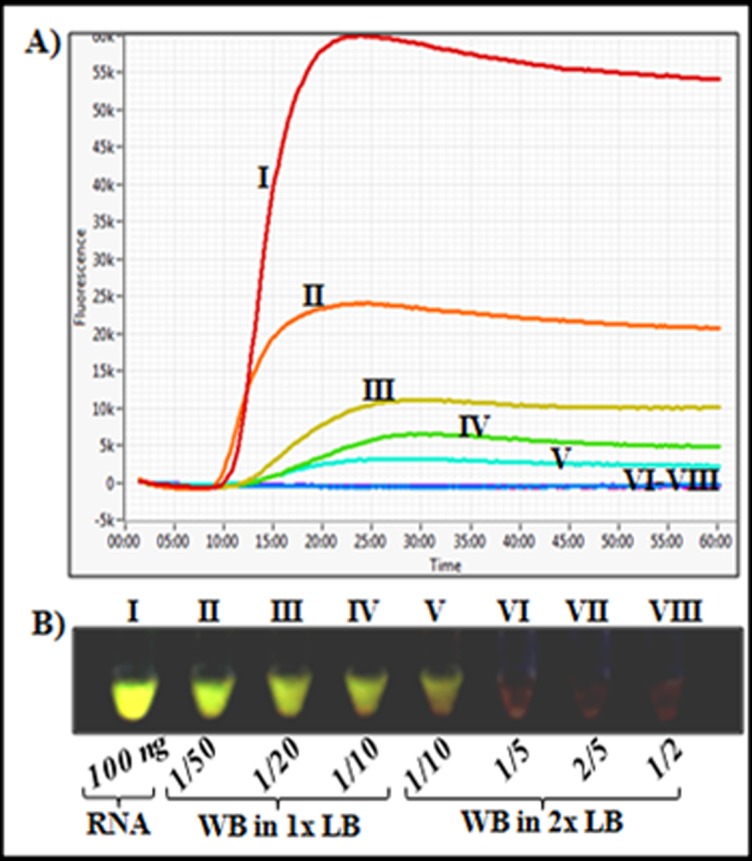
Effect of different blood dilutions on the RT-LAMP signals. One hundred nanograms of total RNA or 2.5 μL of lysed samples from differently diluted (1/2 down to 1/50 in lysis buffer) *P*. *falciparum*-parasitized blood samples were tested by RT-LAMP. **A)** RT-LAMP amplification profiles showing drastic drops in maximum fluorescence intensities with increasing concentration of lysed blood components. **B)** Visual appreciation of GelGreen fluorescence showing masking of fluorescence intensity with increasing concentration of lysed blood components. Whole blood dilutions were as follows; **II**: 1/50, **III**: 1/20, **IV**: 1/10 in 1x lysis buffer, and **V**: 1/10, **VI**: 1/5, **VII**: 2/5, **VIII**: 1/2 in 2x lysis buffer.

**Fig 6:**
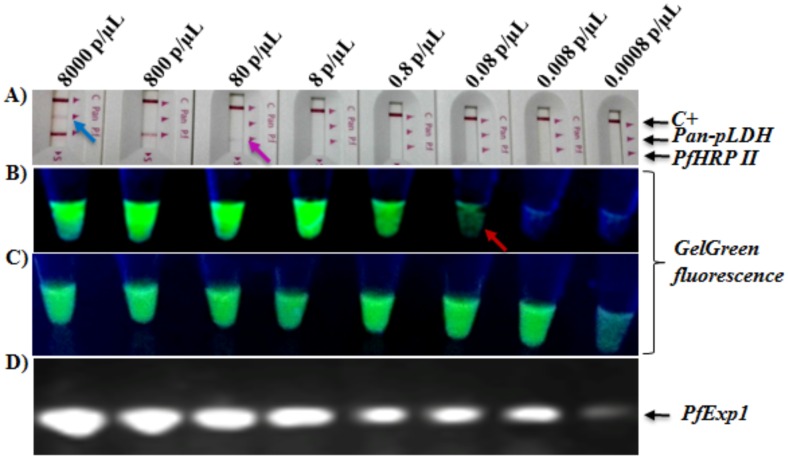
Detection limit of RT-LAMP assays using infected whole blood lysates. **A)**. *P*. *falciparum*-infected blood (8000 parasites/μL) was serially diluted by ten-fold with uninfected whole blood, and 5 μL of each diluted blood sample were also analyzed by RDT for presence of the *P*. *falciparum* species-specific antigen, PfHRP2, and/or positivity for the genus-specific pLDH antigen (pan-pLDH). The parasite densities (parasites/ μL blood) in the tubes prior to testing by RDT are indicated above each reaction tube. C+: RDT positive control band.*Blue* and *purple colored* arrows mark the detection limits of the *P*. *falciparum*-specific (PfHRP II) and pan-*Plasmodium* (pan-pLDH) antigen detection RDTs, respectively. **B)** RT-LAMP assays with 2.5 μL whole blood lysates (10 μL sample in 490 μL lysis buffer) of each sample in the dilution series showing a detection limit of 0.08 parasites/ μL for the whole blood RT-LAMP method (*Red* arrow). **C)** RT-LAMP assays with 2.5 μL extracted RNA (from 2 mL blood volume) from each tube in the dilution series showing a detection limit >0.0008 parasites/ μL for the extracted RNA-based RT-LAMP method. **D)** RT-PCR with2.5 μL extracted RNA (from 2 mL blood volume) from each tube in the dilution series showing a detection limit >0.0008 parasites/ μL for the extracted RNA-based RT-PCR method. Data is representative of the lowest limit of detection obtained from five replicate experiments at different diluted parasite density ranges.

To eliminate the RNA extraction step thereby reducing the assay turnaround time and cost, we investigated the use of whole blood lysates with the developed RT-LAMP method. Ten micro liters of infected whole blood specimen were treated for two minutes at room temperature with lysis buffer (blood dilutions of 1/2 to 1/50 in lysis buffer), and 2.5 μL of each resulting lysates were analyzed directly by RT-LAMP. As shown in Fig 5A and 5B, treatment of the infected samples with the TritonX-100-containing buffer effectively released integral forms of the target that were detected by RT-LAMP. However, as observed in Fig 5A, the maximum fluorescence intensity of the proprietary nucleic acid dye found in the commercialized RT-LAMP mastermix (ISO-DR001,OptiGene, UK), diminished drastically with increasing concentration of blood components in the reaction tubes. A similar masking of fluorescence signals was observed when the nucleic acid dye GelGreen was added to each tube post-amplification and imaged under a UV light box (Fig 5B). These findings suggest a concentration-dependent quenching of the proprietary dye or GelGreen fluorescence by whole blood components, necessitating the use of minute whole blood sample volumes for the RT-LAMP assay. The minimal detection limit of the whole blood lysis method was further determined by serially diluting a microscopically confirmed *P*. *falciparum* mono-infected blood specimen (8000 parasites/μL) down to 0.0008 parasites/ μL in an uninfected whole blood sample followed by rapid lysis (10μL sample in 490μL lysis buffer for two minutes at room temperature), and RT-LAMP as described in the "Methods" section. Five microliters of each serially diluted blood sample were also directly tested for parasite antigen presence using the SD Bioline Malaria Ag *P*. *f*.*/*Pan-*Plasmodium* RDT (Standard Diagnostics) in accordance with the manufacturer's instructions. Meanwhile, total RNA was extracted from two milliliters of each sample in the dilution series and tested by both RT-LAMP and RT-PCR for parasite presence. As represented in Fig 6B and 6C, RT-LAMP detected infection in tubes containing as little as 0.08 parasites/ μL when directly using whole blood lysates, and ~0.0008 parasites/ μL when using RNA extracts. In comparison, the detection limit of the PfHRP2/pan-pLDH antigen-based RDT (SD Bioline) was 80 parasites/ μL for the PfHRP2 band, and ~8000 parasites/ μL for the pan-pLDH antigen band (Fig 6A). These findings suggest that the developed whole blood RT-LAMP method is more sensitive than the above RDT in terms of limits of detection.

### Performance of RT-LAMP assay in NINA heaters

**Fig 7:**
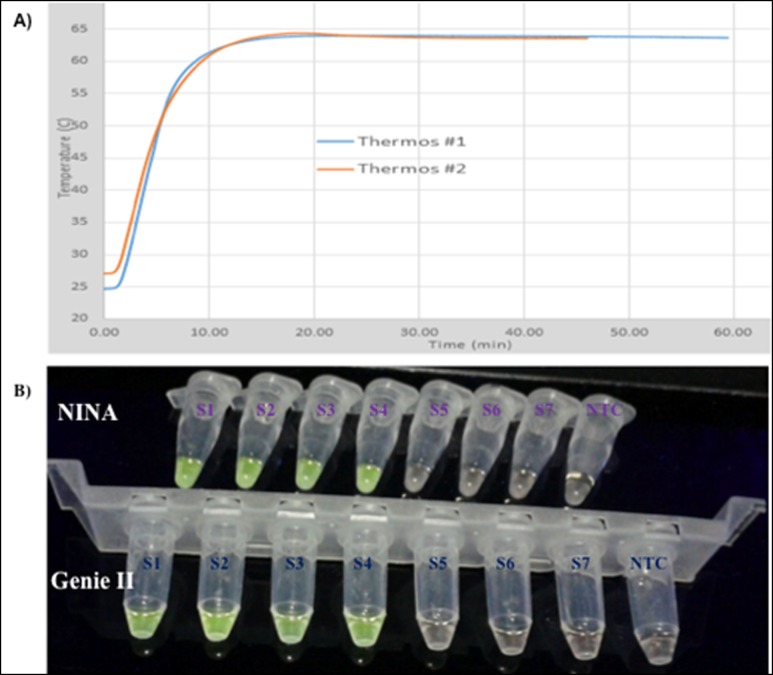
Performance of whole blood RT-LAMP in NINA heaters. Electricity-free NINA heaters were designed taking into account the isothermal temperature requirements of the developed RT-LAMP, and tested under outdoor temperature conditions of 25–32°C. **A)** Temperature profiles of two prototype heaters (Thermos #1 and Thermos #2), showing rapid attainment of isothermal temperature conditions (approximately 65°C) in under 10 minutes, and maintenance of this temperature for up to 60 minutes after heat activation. **B)** Sensitivity limits of NINA and Genie II-based RT-LAMP using serially diluted whole blood lysates in the presence of GelGreen solution. The assays were terminated after 30 minutes of amplification and imaged using a UV light box. The serially diluted parasite densities (parasites/μL) in the reaction tubes (S1 to S7) prior to lysis and RT-LAMP assays were as follows; **S1**: 120, **S2**: 12, **S3**: 1.2, **S4**: 0.12, **S5**: 0.012, **S6**: 0.0012, and **S7**: 0.00012, respectively. **NTC**: No-template control.

To facilitate the use of the whole blood RT-LAMP method in non-electrified field settings, we fabricated two prototype heaters capable of maintaining the required RT-LAMP amplification temperature conditions (65–68°C) for at least 60 minutes under ambient temperatures greater than 25°C. As shown in Fig 7A, the two prototype heaters (Thermos #1 and #2) rapidly attained a maximum temperature of approximately 65°C, when activated at ambient temperatures of 25°C or 27°C, respectively, maintaining this amplification temperature for more than 1 hour. When evaluated in comparison with the Genie II amplifier using serially diluted whole blood lysates in the presence of GelGreen, the same positive reactions were obtained down to a detection limit of 0.12 parasites/ μL whole blood for both the Genie II and NINA heaters (Fig 7B). The same positive and negative results (positivity rate = 82%, concordance = 100%) were obtained when whole blood samples from forty-eight microscopically confirmed cases (mean parasite density: 21,004 parasites/μL, range: 912–70,800 parasites/μL) and twenty negative individuals were analyzed by both NINA and Genie II systems (data not shown). Taken together, the data demonstrate the efficiency of the NINA heaters for use with the developed RT-LAMP method, particularly in resource-limited field settings common in Sub-Saharan Africa.

### Clinical performance of the RT-LAMP method

Clinical evaluation of the RT-LAMP assay was done using extracted RNA for comparisons with RT-PCR, or using whole blood samples for comparison with light microscopy or antigen-based RDT. As shown in Table 2, 70% of the 98 microscopy positive (mean parasite density: 36,986parasites/μL, range: 1660–347,480 parasites/μL) and 34 negative samples were positive by *P*.*f*.-specific RDT (81 *P*.*f/*pan*-*positives and 12 *P*.*f-*only positives), whereas 69% were positive by pan-*Plasmodium* RDT (81 *P*.*f/*pan*-*positives and 10 pan-only positives). The "*P*.*f*.*-*only" positive cases predominantly occurred among the microscopy negative or low-density parasitaemic samples, and are likely to represent RDT "false positives" arising from delayed clearance of the detected PfHRP2 antigen in treated patients. On the other hand, the "pan-only" positives presumably represent non-*falciparum* malaria cases or mixed infections with undetectable levels of *P*. *falciparum* parasitaemias. In fact, six of the pan-only samples were microscopically identified as *P*. *malariae*, three as *P*. *ovale*, and one as a mixed *P*. *ovale* and *P*. *falciparum* infection. Eighty three percent of the 132 samples were positive by RT-PCR, 90%by RT-LAMP using extracted RNA, and 85%byRT-LAMPwith whole blood lysates. Both RT-PCR and RT-LAMP were positive for the single microscopically confirmed *P*. *ovale*/*P*. *falciparum* mixed infection, for one of the six *P*. *malariae* samples, and for one of three *P*. *ovale* samples. These findings suggest that the developed RT-LAMP is specific for *P*. *falciparum* infections, and that the observed positives amongst the microscopically detected *P*. *ovale* and *P*. *malariae* samples are likely to represent mixed infections with submicroscopic *P*. *falciparum* parasite densities.

**Table 2:** Performance of RT-LAMP, RT-PCR and RDT in comparison with thick smear microscopy.

Microscopy		RDT			RT-PCR	RT-LAMP	
Parasites/μl	No. Tested	Pf^+^/Pan^+^	Pf^+^ only	Pan^+^ only	RNA extract	RNA extract	Whole blood
0	34	3	5	-	16	25	21
1–1000	0	0	0	0	0	0	0
1001–5000	18	9	6	2	16	16	13
5001–10,000	26	21	1	4	24	24	24
>10,000	54	48	-	4	54	54	54
Positivity rate	74%	61%	9%	8%	83%	90%	85%

**Fig 8:**
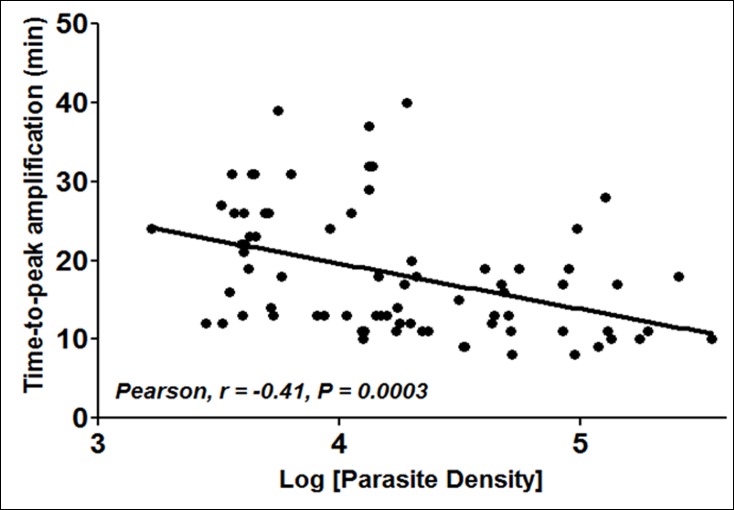
Inverse relation between the whole blood RT-LAMP amplification times and parasite densities. Dot plots of time-to-peak amplification signals of 85 *P*. *falciparum* infected samples and parasite densities (parasites/ μL), showing an inverse correlation (Pearson, r = -0.41, P<0.001) of the RT-LAMP amplification times with blood parasitaemia.

Compared to the reference RT-PCR positives, test sensitivity was 96% (95% CI: 90.95–99.00%) for RT-LAMP using extracted RNA,or90% (95% CI: 82.81–94.90%) for RT-LAMP with whole blood. For the same RT-PCR positives, test sensitivity was 81%(95% CI: 72.31–87.7%) for the *P*.*f*-specific RDT,81%(95% CI: 72.31–87.78%) for pan-*Plasmodium* RDT, and 85% (95% CI: 77.46–91.45%) for thick blood smear microscopy. Assay specificities were 41% (95% CI: 20.71–63.65%) for RT-LAMP with extracted RNA or whole blood lysates, 82% (95% CI: 59.72–94.81%) for the *P*.*f*.*-*specific RDT, 91% (95% CI: 70.84–98.88%) for pan-positive RDT, and 82% (95% CI: 59.72–94.81%) for thick blood smear microscopy. The concordance rates between the different methods are presented in Table 3, indicating no significant difference (McNemar's test, P>0.05) betweenRT-PCR and RT-LAMP, microscopy and RDT, or between RT-LAMP with extracted RNA and RT-LAMP with whole blood lysates. Weak concordance rates were obtained between each RT-LAMP or RT-PCR method and light microscopy or RDT **(**Table 3). Consistent with data using RNA extracts (Fig 2C), an inverse correlation (Pearson r = -0.41, P = 0.0003) was observed between the whole blood RT-LAMP peak amplification times and parasite densities (Fig 8), highlighting the potential usefulness of the whole blood RT-LAMP method in quantifying the infection. Together, the data indicate equivalence of the RT-LAMP and RT-PCR methods, and superiority of both methods over RDT or thick blood smear microscopy in terms of test sensitivity.

**Table 3:** Assay concordance rates and statistical significance.

P values (McNemar’s test)	Microscopy	Pf-RDT	Pan-RDT	RT-PCR	RT-LAMP (RNA Extracts)	RT-LAMP (Whole blood)
Microscopy	1	84%	90%	85%	78%	79%
Pf-RDT	**0.3827**	1	83%	81%	79%	84%
Pan-RDT	**0.0961**	**0.8312**	1	83%	78%	77%
RT-PCR	0.0139	0.0014	0.0002	1	87%	82%
RT-LAMP (RNA Extracts)	0.0002	0.0001	0.0001	**0.0523**	1	91%
RT-LAMP (Whole blood)	0.0140	0.0001	0.0003	**0.8383**	**0.0704**	1

## Discussion

We report here the development of a simple, rapid, highly sensitive and specific RT-LAMP method for *P*. *falciparum* malaria diagnosis that is based on the detection of high abundant transcripts in extracted RNA, or directly in whole blood lysates.

To obtain test sensitivities needed to detect low-level parasite carriages common in malaria endemic communities worldwide, high-abundant and high copy number RNA transcripts were targeted, resulting in a detection limit of 0.25ng total RNA per 25μL RT-LAMP or RT-PCR reaction, and as little as 0.08 parasites/ μL for the RT-LAMP approach when using whole blood lysates. Indeed, seminal studies by Kamau *et al* and Murphy *et al* have previously demonstrated the benefits of including an RT step in molecular diagnosis for malaria[40, 41]. While targeting the *Plasmodium* 18S rRNA/DNA sequence, the authors reported a 10-fold increase in assay sensitivity when qRT-PCR was used instead of qPCR for the genus-specific test, and 3-fold increase in the case of *P*. *falciparum*-specific detection. A major advantage of LAMP-based approaches over PCR methods is in their rapid turnaround times (time-to-positive signal), simplicity, and insensitivity to common PCR inhibitors[48, 64–66]. Due to difficulties in obtaining sufficiently specific LAMP primers targeting the 18S rRNA transcript, the *Plasmodium* genome database was searched for other highly expressed genes, resulting in the identification of the *PfExp1* open-reading frame as an excellent diagnostic target. By using the Genie II LAMP amplifier (OptiGene, Ltd) and a commercially available single reverse transcriptase/strand displacement DNA polymerase enzyme mix (ISO-DR001, OptiGene, Ltd), positive RT-LAMP signals were detected as early as 8 minutes into the reaction and in most cases saturating after 20 minutes of reaction time. This increase in amplification speed, when compared to the usual 30–40 minutes time-to-peak amplification of some previously reported DNA-based malaria LAMP assays[50, 67, 68] may be attributed to:**1**) the higher copy number of RNA transcripts compared to the DNA targets, **2**) the high abundance of the *PfExp1* transcript (minimum transcript expression percentile of 98%)[61], **3**) the high reverse transcription/polymerase efficiency of the proprietary enzyme in the ISO-DR001 mastermix (OptiGene, Ltd), and **4**) the optimized primer and temperature conditions in this study.

To eliminate the RNA isolation step and shorten assay turnaround time and cost, a simple “lyse and amplify” protocol was designed and optimized. This allowed processing of patients samples in just two minutes, followed by less than forty minutes reaction time to record a positive signal. Indeed, this study is the first report of a LAMP-based method, targeting parasite RNA instead of DNA in blood lysates as reported previously [66, 69]. A limitation of the whole blood lysis method presented in this study is in the large volume of lysis buffer needed to achieve an appropriate dilution (1:50) of the blood components, which appear to exhibit fluorescence quenching effects on the nucleic acid dyes used. However, the inherent large copy number of the *PfExp1* target and the sensitivity of the transcript-based RT-LAMP method overcome the impact of specimen dilution, allowing the detection of approximately 0.08 parasites/ μL in less than 30 minutes of reaction time when directly testing 10 μL of whole blood samples. An even lower limit of detection (~0.0008 parasites/ μL) was obtained when RNA was extracted from standardized two milliliters blood samples, and 5% of the extract analyzed by RT-LAMP or RT-PCR. Compared to current DNA-based LAMP tests for malaria with detection limits of 1–10 parasites/ μL [44, 45, 68, 70], the RT-LAMP method described in this study represents a major improvement in malaria diagnostics that will allow for identification of low-level parasite carriages common in malaria endemic countries.

Both RT-PCR and RT-LAMP outperformed conventional microscopy and RDT methods in terms of test positivity rates when analyzed on samples from 132 suspected malaria cases. Additionally, the RT-LAMP method exhibited higher clinical sensitivity than RDT and thick blood smear microscopy, which were negative in up to 30% and 26% of the study population, respectively. Although the clinical specificity of the newly developed assay could not be determined reliably in the study population (febrile malaria endemic residents), it seemed unlikely that the observed higher sensitivity of both molecular tests were due to non-specificity of the primers used or to cross-contamination of negative samples during the experiments. Indeed, melting curve analyses and assays with RNA extracts or whole blood samples from microscopically confirmed non-*P*. *falciparum* infections (nine *P*. *malariae* and five *P*. *ovale* overall) demonstrated clearly that the developed RT-LAMP method was specific to *P*. *falciparum*. Such species-specificity of the assay might arise from the shown differences between the *PfExp1* sequence and other *Plasmodium Exp1* sequences, particularly within the RT-LAMP primer sites (Fig 1), as well as the requirement for at least four of the six primers to bind cognate sites within the targeted sequence to yield positive RT-LAMP signals.

Overall, a good agreement (concordance rates >75%) was observed between the different methods, consistent with the high parasitemia levels of the study population (mean parasite density:36,986 parasites/μL, range: 1660–347,480 parasites/μL). However, such agreements between the different tests are likely to vary between populations of different infection intensities, given the reported differences in the test sensitivity limits. Contrarily to data showing a strong negative correlation (Pearson r = -0.9777, P = 0.004) between the RT-LAMP amplification times and target RNA amounts, only a moderate correlation (Pearson r = -0.41, P = 0.0003) was observed between parasite densities in clinical samples and the amplification times. Such differences in correlation coefficients may be attributed to the fact that mixed *Plasmodium* spp infections were common in the study population, and because microscopically measured parasite densities in mixed infections do not represent actual *P*. *falciparum* parasitaemia needed for association studies with the species-specific RT-LAMP data. The RT-LAMP assay was positive in thirteen of twenty-two RT-PCR negative samples, giving a relative clinical specificity of only 41% for the RT-LAMP assay. Considering that seven of these RT-PCR negatives were also positive by microscopy and/or RDT, it seemed unlikely that the thirteen RT-LAMP positives were "false-positives" of the new method. A repeat analysis of all twenty-two RT-PCR negatives yielded the same results, suggesting that such cases represented RT-PCR "false-negatives", which might result from presence of unwanted RT-PCR inhibitors in the samples. Indeed, despite exhibiting the same detection limit as RT-LAMP, the RT-PCR method was positive in slightly fewer number of the clinical samples than RT-LAMP, suggesting the presence of RT-PCR inhibitors in some of the samples.

To further adapt the RT-LAMP system for field use, particularly in high ambient temperature and resource-limited settings common in sub-Sahara Africa, we designed electricity-free and portable heating devices as replacement for the Genie II amplifier. This NINA system relies on the use of commercially available and inexpensive (US$0.06 per test) MgFe fuel packs and common salt solution to generate heat inside a reusable PCM-based housing[51, 60]. The prototype heaters developed in this study performed similarly to the Genie II LAMP amplifier, the single draw back being its dependence on end-point visualization of the positive outcomes using commercially available UV flashlights. Taken together, the RT-LAMP method described in this study provides significant improvements in malaria molecular diagnosis in terms of test sensitivity, field applicability, assay turnaround time, and potential cost-effectiveness. The demonstration that increased diagnostic sensitivity can be achieved by reverse transcription-based techniques targeting high-abundance RNA transcripts is of broad significance.
